# Clinical predictors of psychotropic medication prescription in children with ASD of the ELENA cohort

**DOI:** 10.3389/fpsyt.2023.1153543

**Published:** 2023-07-21

**Authors:** Marie-maude Geoffray, Matias Baltazar, Cécile Michelon, Lucie Jurek, Amaria Baghdadli

**Affiliations:** ^1^Centre Hospitalier Le Vinatier, Bron, France; ^2^Université Claude Bernard Lyon 1, Lyon, Rhône-Alpes, France; ^3^INSERM U1290 Recherche sur la Performance des Soins (RESHAPE), Lyon, Rhône-Alpes, France; ^4^Université de Montpellier, Montpellier, Languedoc-Roussillon, France; ^5^Centre Hospitalier Universitaire de Montpellier, Montpellier, Languedoc-Roussillon, France; ^6^Centre de Ressources Autisme Languedoc-Roussillon, Montpellier, Languedoc-Roussillon, France

**Keywords:** autism, psychotropic, children, ASD, predictors

## Abstract

Psychotropic drugs are often used to treat behavior problems in ASD with some evidence supporting efficacity (e.g.: risperidone and irritability) but also significant side effects at the short and longer-term. It is then essential to know better the factors associated with the prescription of these medications and potentially implement early behavioral and psychosocial intervention or cognitive remediation before to use medication. We designed a case–control study based on the population of the ELENA cohort to assess the factors associated with early psychotropic drugs use in children with ASD. Externalized behavior symptoms (measured by the Child Behavior Checklist) is the leading risk factor during the first years of follow-up (aOR = 2.8; CI [1.04; 7.67]; *p* = 0.04). Age, gender, autism severity, adaptive behaviors, or internalized behaviors were not associated with psychotropic medication prescription. Low IQ and parents who had received training tended to increase the risk of psychotropic medication prescription during follow-up but were not statistically significant. These findings underscore the need for early identification of symptoms of externalizing behaviour, early appropriate information for parents about treatment with and without medication, early analysis of externalising behaviour and targeted treatments.

## Introduction

Autism spectrum disorder (ASD) is a neurodevelopmental disorder characterized by impaired social communication and interaction and unusually restricted, repetitive patterns of behavior and interests (1). About 50% of children with ASD exhibit behavioral problems which interfere with the performance of daily living skills and may increase social isolation (2).

Psychotropic drugs are often used to treat behavior problems in ASD (3–5), with some evidence supporting efficacity (e.g.: irritability) but also significant side effects at short and longer-term (3–7). For these reasons, it is essential to know better the factors associated with the prescription of these medications and potentially implement early behavioral intervention or remediation cognitive and delay or avoid psychotropic drugs.

To date, most studies on psychotropic prescription in individuals with ASD have been descriptive in nature, with very few examining the factors associated with the use of psychotropic medication (8). Houghton and colleagues explored predictors of psychotropic medication in people with ASD 3 years old and up (4). The authors found an increase in prescriptions with age. Surprisingly, they found that all types of psychiatric comorbidities increased the odds of receiving any psychotropic medication, except for conduct disorder. In 2017, Rasmussen and colleagues showed that children with comorbid ADHD and intellectual disability were the most frequently medicated [around 60%; (5)]. The impact of sex, IQ level, or autism severity have also been evaluated, but there are substantial discrepancies between studies on the effect of these factors (7).

There is a lack of clinical studies regarding this topic in the scientific literature. Most of the studies are based on health-insurance data, which allows the analysis of socio-demographic variables, but without thorough clinical phenotyping of participants, which is relevant to assess potential risk and protective factors. Moreover, most of the related studies are performed in North America. Few European studies are available, although prescription rates are highly different from US and Canadian (7). To our knowledge, there is no French study on this topic.

The objective of our study was to assess the potential medico-psycho-sociological protective and risk factors associated with early psychotropic drugs use in children with ASD, based on a French cohort of individuals who received a thorough psychiatric and psychometric assessment. A case–control study was performed based on data from a large cohort of children diagnosed with ASD: the ELENA cohort, an open, multicenter, longitudinal, prospective study. Incident cases of psychotropic drug use in children with ASD were compared to a control population. According to the recent literature, we hypothesized an increase in psychotropic drugs prescription with the presence of psychiatric comorbidities, lower IQ, and higher age and decrease in prescription when the parents are better informed about ASD and behavioral problem.

## Methods

### Participants

The present study is based on a subset of data from a large French multiregional prospective, cohort [ELENA; (9)].^1^ All the investigator centers of ELENA are specialized in assessment for ASD. Children and their parents are coming to these centers for diagnosis. After providing information to parents and obtaining their consent, ASD participants were included between 2 and 16 years of age and with an ASD diagnosis established by a multidisciplinary clinical assessment based on the DSM-5 criteria (10). The clinical evaluation comprised the Autism Diagnostic Observation Schedule 2 [ADOS-2; (10)], the Autism Diagnostic Interview-Revised [ADI-R; (11)], a parental interview concerning the child’s adaptive functioning [VABS-II; (12)], and psychological examinations to assess IQ. Thirteen French autism research centers participated in the inclusion of patients. After inclusion, patients are reassessed every 18 months, starting with V0 (assessment at inclusion). Reassessments are still ongoing in this cohort (up until 4 years after inclusion), so we focused on the visits with the most completion rate to this date, meaning baseline (V0), V1 (V0 + 18 months), and V2 (V0 + 36 months).

To evaluate the risk factors of the introduction of psychotropic medication during the follow-up, we included participants of the cohort who did not receive any psychotropic medication at baseline (V0) and who completed at least one of the two first follow-up visits (i.e., we only included participants who completed V0 and additionally completed either V1 or V2 or both). The introduction of a psychotropic medication defined cases during the follow-up (at V1 or V2). Children who did not receive any psychotropic medication during the follow-up were considered control participants. We selected children without psychotropic medication at baseline to ensure that exposure factors precede the treatment prescription.

### Measures

The Autism Diagnostic Observation Scale, second version (ADOS-2) was used for diagnosis but also for the measure of autism evolution and severity. The comparison and severity score (ADOS CSS) varied from 1 to 10; the higher the score, the more severe the autistic symptomatology.

The intellectual level was measured with the ‘Best Estimate’ IQ score according to the literature in children with intellectual disabilities (13, 14). The ‘Best estimate’ IQ score was derived based on the cognitive test that was deemed the more appropriate regarding each participant’s age and verbal ability. Wechsler standardized scores were preferred when available (using the Perceptual Reasoning Index, the Fluid Reasoning Index, or the Performance IQ, depending on the version). For other tests which provide scores that are not standardized in the same way, we computed developmental quotients [for example, using the Cognitive Verbal/Preverbal index of the PEP-3: CVP/chronological age*100; see (13, 14)] for similar methodology. ‘Best estimate’ IQ average score is in the general population of 100 with a standard deviation of 15. Higher is the ‘Best estimate IQ’, better is the intellectual level. A ‘best estimate’ IQ score <70 is in favor of an intellectual development disorder.

Adaptive functioning was measured with the Vineland Adaptive Behavior Scale 2nd Edition (15). The VABS-II is a standardized, semi-structured interview administered to parents. The VABS-II includes domains of communication, daily living skills and socialization. It is validated (15) and used in numerous autism studies. The global normalized score and the 3 subscales have a mean of 100 (SD = 15). The higher the score, the better is adaptive functioning.

Externalizing and internalizing behavioral problems were assessed using the Child Behaviour Checklist [CBCL; (16)], a standardized validated, caregiver report exploring emotional and behavioral problems in children and adolescents (17). The CBCL provides two scales: internalizing and externalizing problems. The internalizing scale consists of five subscales: Anxious/Depressed, Withdrawn/Depressed, Somatic Complaints, Social Problems, and Thought Problems. The externalizing scale consists of three subscales: Rule-Breaking Behaviour, Attention Problems, and Aggressive Behaviour. Normalized scores for each scale (internalizing and externalizing problems) can be obtained, considering age and biological sex. T-scores <60 are regarded as within the normal range, whilst *t*-scores of 60–64 indicate borderline clinical problems, and t-scores ≥64 indicate marked clinical problems. The higher is the score, the higher are the problems.

We measured the parents’ stress levels directly associated with their parenting role using the parenting Stress Index, Fourth Edition, Short Form [PSI™-4-SF; (18)]. This validated questionnaire is self-administered, takes approximately 10 mn to complete, and allow for assessing three components: the level of stress related to (1) parental distress, (2) the perception of the child as being “difficult,” and (3) dysfunctional interactions between the child and the parent. A total stress score is measured. Higher is the score, higher is the parental stress.

The parent training variable was self-declarative. The parent answered the question: “Have you ever received training on ASD?” They answered “yes” or “no.” No additional information was requested in this cohort.

### Statistical analyses

Children were divided into two groups regarding psychotropic drug use: with psychotropic drug(s) during the follow-up (i.e., cases) versus without the psychotropic medication(s) during the follow-up (i.e., controls).

Univariate analyses were used to examine associations between dependent variable [psychotropic drug use (2 groups)] and potential risk factors [socioeconomic status (high vs. middle vs. low)], children’s characteristics (age: 6yo + vs. 5yo-; biological sex: male vs. female; best-estimated IQ: 69− vs. 70+; CBCL Internal: T score of 60+ vs. 59−; CBCL External: T score of 60+ vs. 59−) and family antecedents [family structure: one-parent vs. both-parents family; parents self-declared training: present vs. absent; parental stress assessed by the Parental Stress Index dichotomized in 85+ vs. 84−; (18)]. We used the parametric Student t-tests or non -parametric Mann–Whitney test or Kruskal–Wallis for continuous variables depending on the normality of the distributions assessed by the Shapiro–Wilk Test. The Chi-square test was used to compare categorical variables, and Fisher exact test was used in case expected values within cells were inferior to five. Pairwise comparisons were made using the Bonferroni *post-hoc* test to correct for multiple comparisons.

In accordance with Hosmer [2013], univariate analyses were used to identify potential candidates for the multivariable model at an alpha level = 0.2.

The outcome and all variables with a value of *p* < 0.20 for association were entered in the final model. We used multivariable logistic regression to determine what factors were associated with psychotropic drug use while controlling for other factors (age at baseline and biological sex).

Odds ratios (OR) and 95% confidence intervals (CI) are reported. The goodness-of-fit of the models was assessed using the Hosmer and Lemeshow test.

The significance level used was 5%. Statistical analyses were performed using SAS version 9.3 (SAS Institute, Cary, North Carolina).

## Results

### Sample

**Figure 1:**
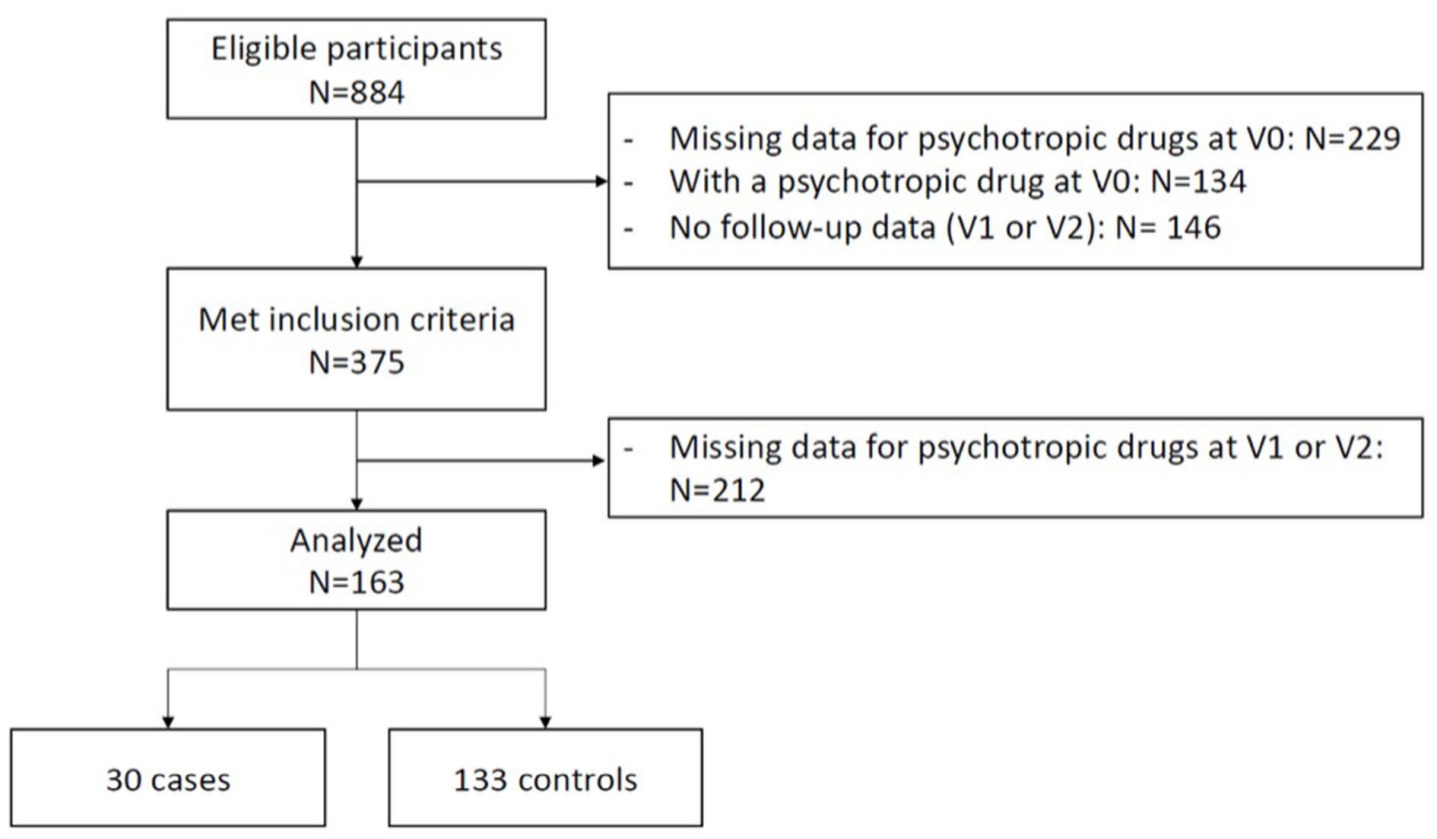
Flowchart presenting the final sample.

The flowchart presenting the final sample is displayed in Figure 1. Of the 884 eligible participants, 375 met the inclusion criteria. Of the 375 participants that met the inclusion criteria, 212 were excluded from the analyses because of missing psychotropic medication status at follow-up (i.e., missing data for our main outcome). The first analyses were processed with 30 cases and 133 controls.

The supplemental file presents a description of the excluded participants because of psychotropic medication or missing baseline data (Supplementary Table S1). There were 229/884 (25.9%) participants with missing data regarding whether or not they were taking medication at baseline, and 177/655 (27%) of the cohort’s participants with available data had a prescription for psychotropic medication and were thus excluded. Excluded participants who were already taking medication were significantly older than participants without psychotropic medication or those excluded because of missing data and had significantly lower adaptive levels of functioning in socialization. No difference concerning biological sex or ADOS CSS was found.

The characteristics at baseline of included cases and controls can be found in Table 1.

**Table 1:** Cases and controls characteristics: description of the two populations (not receiving or receiving medication during the 3 following years) at inclusion.

Group	Controls	Cases	Statistic	*p* value
	*N* = 133	*N* = 30		
Number of females (%)	26 (19.5)	6 (20.0)	Chi-square test	0.96
Mean age in years (SD; range)	5.66 (3.02; 2.0–14.9)	5.54 (2.98; 2.0–12.5)	Mann–Whitney test	0.87
Mean best estimate IQ (SD; range)	75.3 (26.7; 20–138) *n* = 118	72.6 (31.4; 20–125) *n* = 27	Student test	0.65
Mean ADOS calibrated severity score (SD; range)	6.54 (1.94; 1–10) *n* = 111	7.27 (1.73; 4–10) *n* = 26	Mann–Whitney test	0.097
**VABS-II mean domain scores (SD; range)**				
Communication	71.9 (14.2; 38–108)	67.2 (12.5; 36–89)	Student test	0.099
Daily living skills	72.0 (11.0; 38–104)	69.1 (7.3; 56–86)	Mann–Whitney test	0.24
Socialization	75.5 (12.7; 30–95) *n* = 132	73.5 (8.3; 45–82) *n* = 30	Student test	0.41

SD, standard deviation. Variables are described by their number, percentage, mean, standard deviation, and range. The *n* for each analysis are specified (ns) when different from the overall *N* of the group.

All participants (cases + controls) in the present study had a mean age of 5.7 years, with a 1:4.1 female to male sex ratio, and 41.4% of participants with low intellectual functioning (i.e., IQ < 70). Cases and control participants were not different in sex ratio, age, intellectual functioning, ADOS severity score, and Vineland-II domain scores. The type of psychotropic drugs taken by the participants can be found in Table 2.

**Table 2:** List of reported psychotropic treatments in cases (that is children receiving medication during the following after inclusion).

Class	INN	Number of patients with medication (% of cases)
Psychostimulants	Methylphenidate chlorydrate	7 (23%)
Antipsychotics	Risperidone	5 (17%)
	Aripiprazole	1 (3%)
Antiseizure	Valproate de sodium	2 (7%)
	Clonazepam	1 (3%)
Benzodiazepine	Midazolam chlorydrate	1 (3%)
Antidepressant		0 (0%)
Others	Melatonine	16 (53%)
	Hydroxyzine chlorydrate	5 (17%)

INN, international nonproprietary name. Namely, 1 case may have received several different drugs.

### Results of analyses

Univariate analysis showed a significative association between psychotropic drug use and “Parental stress index,” “External behavior T-Score,” “parent training,” and “Best estimate IQ.”

There were many missing data for the parental stress index (45% of missing data for controls leading to only 22 cases and 68 controls). Therefore, we decided to drop this variable from final regression model, even though this variable was significantly different in controls and cases in the univariate model (*p* = 0.03). When dropping this variable, we managed to keep a sample of 25 cases and 97 controls.

The regression analysis shows that participants with an externalization score of more than 60 at baseline (clinical threshold) have a significantly increased risk of being medicated with a psychotropic drug at either V1, V2, or both, when controlling for another covariate such as biological sex, age at baseline, (aOR = 2.8; CI [1.04; 7.67]; *p* = 0.04; Table 3). We also found a marginal effect for parents’ self-declared training in ASD, with a higher risk for parents who have received training. Finally, we found a marginal effect of intellectual functioning, with participants with an IQ lower than 70 at baseline with higher risks of receiving psychotropic medication. No other variables were found to be significant in the logistic regression.

**Table 3:** Multivariable logistic regressions.

	(cas = 25/controls = 97)	
Variable	aOR of treatment (95%CI)	Value of *p*
External T-score (>60 vs. ≤60)	2.8 (1.04; 7.67)	0.04
Training (Yes vs. No)	2.3 (0.9; 5.8)	0.088
Best IQ (<70 vs. ≥70)	2.3 (0.9; 5.9)	0.077

aOR, adjusted odds ratio; included variables: external T-score, training and best IQ; excluded variables: internal T-score; covariates: biological sex and age at baseline.

## Discussion

In this case–control study based on the population of the ELENA cohort, we demonstrate that presenting high externalized behavior symptoms (i.e., with a score higher than 60 at the CBCL externalizing problems scale) at inclusion is the main risk factor for children with ASD for receiving a psychotropic medication during the follow-up. Age, gender, autism severity, adaptive behaviors, or internalized behaviors were not associated with psychotropic medication prescription. Low IQ and parents who had received training on ASD tended to increase the risk of psychotropic medication prescription during follow-up, but these associations were not statistically significant.

A significant implication of our findings is that high externalizing behavior, Rule-Breaking Behaviour, Attention Problems, and Aggressive Behaviour is the main facilitator for introducing psychotropic drugs in children with ASD. They are easy to identify and may offer the possibility of implementing recommended early behavioral interventions or cognitive remediation targeting these externalizing behaviors. A well-conducted behavioral intervention, an adapted protocol set up for the targeted behavior, or cognitive remediation is recommended before a psychotropic drug is introduced. Such interventions may avoid over-prescription in the follow-up (2, 19, 20).

Parent training often includes modules to manage the child’s externalized behaviors. Thus, we might expect a decrease in externalized behaviors when parents are more educated. In fact, we could hope that these parents ask or look for targeted behavioral intervention or cognitive remediation. However, contrary to our expectations, even if this finding did not reach statistical significance, having a parent “trained in ASD” tended to be associated with psychotropic medication prescription at follow-up in this study. It is possible that parents receiving psychoeducation or similar training learn about medication that could be prescribed for ASD and are more prone to ask for medicine when encountering difficulties with their children. Drug treatments are often more rapid in their effect than behavioral interventions or cognitive remediation, and also more accessible, contrary to well-trained professionals specialized in intervention to decrease externalized behavior symptoms. We could then expect that targeted parent training may facilitate early and recommended use of medicine to treat attention deficit disorder with or without hyperactivity or sleep disorders, for instance, and fewer antipsychotics. Future longitudinal studies with a more significant sample could evaluate the type of psychotropic medication prescribed (e.g., Melatonin and methylphenidate versus risperidone) in children.

It’s worth to note in our study the parent training variable was self-declarative, and the parent answered only the question, “Have you ever received training on ASD?.” These training can be very different from each other. They can range from a few hours to several days of training with practical application. Above all, they can be different, ranging from general training (“psychoeducation”) to more targeted training in challenging behavior. The training can also be differentiated according to whether the parents are required to implement an intervention in the child’s living environment (2). Future longitudinal studies will benefit from reporting data about the training’s type, intensity, and duration and refining the understanding of the impact of parent training on prescribing medication and behavioral change.

Age was not associated with the subsequent prescription of psychotropic medication in our study. Our age range at baseline was relatively young and narrow compared to previous studies, which often include individuals of various ages [for example, 3–50+ years old in Houghton and colleagues’ work; (4)]. The design of our study can explain this difference as we excluded participants who were already receiving treatment at VO. These participants were significantly older by 1 year than the children without medication at VO (*p* < 0.01).

Our study suggests that prescription practices in France conform to international recommendations more than before (21). In a French self-declared retrospective study analyzing data collected in 2005–2007 (22), Cravero and colleagues showed that the prescription of psychostimulants and melatonin was almost nonexistent at the time (4% of a 393 ASD sample for psychostimulants), while antipsychotics were the most prescribed (23%). In comparison, in our sample, melatonin and methylphenidate were the two most prescribed medications for our case participants.

An important part of the eligible population had missing data for our outcome of interest (psychotropic medication prescription status at follow-up), which can be associated with selection bias. However, a comparison of the missing data population and the eligible population was presented, with minimal differences. Notably, the studied population was retrieved from a cohort of well-diagnosed children with ASD with complete data for essential characteristics such as autism severity, IQ, and adaptive behavior. The data were prospectively retrieved, which avoided the risk of recall bias. We selected children without psychotropic medication at baseline to ensure that exposure factors precede the psychotropic medication prescription.

Our results that the introduction of a psychotropic drug predicts externalized behavioral problems have strong implications for clinical practice in terms of their early screening and specific management. This underlines the importance for clinicians to guide parents as early as possible on the different limitations, side effects and benefits of drug and non-drug treatments for the management of externalized behavioral disorders in order to make an informed and shared decision. But above all, all parents should be systematically trained to identify and manage the first symptoms (e.g., slight intolerance to frustration, infrequent opposition, sleeping problems) of externalized behavior disorders before they become too entrenched, exhaust the child’s environment, and potentially stablish a negative feedback loop between the child and the caretakers.

## Data availability statement

The original contributions presented in the study are included in the article/Supplementary material, further inquiries can be directed to the corresponding author.

## Ethics statement

The studies involving human participants were reviewed and approved by Baghdadli et al. (9), http://www.elena-cohorte.org/. Written informed consent to participate in this study was provided by the participants’ legal guardian/next of kin.

## Author contributions

M-mG, MB, CM, LJ, and AB contributed to the study’s conception and design. AB is the PI of the ELENA cohort. LJ and CM performed material preparation, data collection, and analysis. M-mG, LJ, MB, and AB wrote the first draft of the manuscript. All authors contributed to the article and approved the submitted version.

## Funding

This research received support from the French Health Ministry (DGOS) and CNSA. The CHU of Montpellier (AOI) provided additional support. The funders had no role in study design, data collection, analysis, publishing decisions, or manuscript preparation.

## Conflict of interest

The authors declare that the research was conducted in the absence of any commercial or financial relationships that could be construed as a potential conflict of interest.

## Publisher’s note

All claims expressed in this article are solely those of the authors and do not necessarily represent those of their affiliated organizations, or those of the publisher, the editors and the reviewers. Any product that may be evaluated in this article, or claim that may be made by its manufacturer, is not guaranteed or endorsed by the publisher.

## Footnotes

^1^ http://www.elena-cohorte.org/

## References

[1] American Psychiatric Association. Diagnostic and Statistical Manual of Mental Disorders. 5th Edn. Washington, DC: American Psychiatric Publishing (2013).

[2] Bearss K Johnson C Smith T Lecavalier L Swiezy N Aman M. Effect of parent training vs parent education on behavioral problems in children with autism Spectrum disorder. JAMA. (2015) 313:1524–33. doi: 10.1001/jama.2015.3150, PMID: 25898050 PMC9078140

[3] Fusar-Poli L Brondino N Rocchetti M Petrosino B Arillotta D Damiani S. Prevalence and predictors of psychotropic medication use in adolescents and adults with autism spectrum disorder in Italy: a cross-sectional study. Psychiatry Res. (2019) 276:203–9. doi: 10.1016/j.psychres.2019.04.013, PMID: 31102885

[4] Houghton R Liu C Bolognani F. Psychiatric comorbidities and psychotropic medication use in autism: a matched cohort study with ADHD and general population comparator groups in the United Kingdom. Autism Res. (2018) 11:1690–700. doi: 10.1002/aur.2040, PMID: 30380202

[5] Rasmussen L Bilenberg N Thomsen Ernst M Abitz Boysen S Pottegård A. Use of psychotropic drugs among children and adolescents with autism Spectrum disorders in Denmark: a Nationwide drug utilization study. J Clin Med. (2018) 7:339. doi: 10.3390/jcm7100339, PMID: 30308952 PMC6211111

[6] Downs J Hotopf M Ford T Simonoff E Jackson RG Shetty H. Clinical predictors of antipsychotic use in children and adolescents with autism spectrum disorders: a historical open cohort study using electronic health records. Eur Child Adolesc Psychiatry. (2016) 25:649–58. doi: 10.1007/s00787-015-0780-7 26472118 PMC4889626

[7] Jobski K Höfer J Hoffmann F Bachmann C. Use of psychotropic drugs in patients with autism spectrum disorders: a systematic review. Acta Psychiatr Scand. (2017) 135:8–28. doi: 10.1111/acps.12644, PMID: 27624381

[8] Lake JK Weiss JA Dergal J Lunsky Y. Child, parent, and service predictors of psychotropic polypharmacy among adolescents and young adults with an autism Spectrum disorder. J Child Adolesc Psychopharmacol. (2014) 24:486–93. doi: 10.1089/cap.2014.0011, PMID: 25329798

[9] Baghdadli A Miot S Rattaz C Akbaraly T Geoffray M-M Michelon C. Investigating the natural history and prognostic factors of ASD in children: the multicEntric longitudinal study of childrEN with ASD - the ELENA study protocol. BMJ Open. (2019) 9:e026286. doi: 10.1136/bmjopen-2018-026286, PMID: 31221874 PMC6588969

[10] Lord C. Rutter M. DiLavore P. C. Risi S. Gotham K. Bishop S. L.. Autism diagnostic observation schedule. 2nd Edn. Torrance, CA: Western Psychological Services (2012).

[11] Lord C Rutter M Le Couteur A. Autism diagnostic interview-revised: a revised version of a diagnostic interview for caregivers of individuals with possible pervasive developmental disorders. J Autism Dev Disord. (1994) 24:659–85. doi: 10.1007/BF02172145, PMID: 7814313

[12] Sparrow SS Cicchetti DV Balla DA. Vineland Adaptive Behavior Scales:(VABS) NCS Pearson (2005).

[13] Dellapiazza F Michelon C Picot M-C Baghdadli A. A longitudinal exploratory study of changes in sensory processing in children with ASD from the ELENA cohort. Eur Child Adolesc Psychiatry. (2021) 31:1–10. doi: 10.1007/s00787-021-01746-1, PMID: 33660026

[14] Howlin P Savage S Moss P Tempier A Rutter M. Cognitive and language skills in adults with autism: a 40-year follow-up. J Child Psychol Psychiatry. (2014) 55:49–58. doi: 10.1111/jcpp.12115, PMID: 23848399

[15] Sparrow SS Cicchetti DV Balla D. Vineland Adaptive Behavior Scales, Second Edition (Vineland™-II) Pearson (2005).

[16] Rescorla L. Manual for the ASEBA School-Age Forms & Profiles: An Integrated System of Multi-Informant Assessment ASEBA, Research Center for Children, Youth and Families (2001).

[17] The Research Network on Youth Mental Health Ebesutani C Bernstein A Nakamura BJ Chorpita BF Higa-McMillan CK. Concurrent validity of the child behavior checklist DSM-oriented scales: correspondence with DSM diagnoses and comparison to syndrome scales. J Psychopathol Behav Assess. (2010) 32:373–84. doi: 10.1007/s10862-009-9174-9, PMID: 20700377 PMC2914253

[18] Abidin RR. PSI™-4-SF Parenting Stress Index™. 4th ed. Short Form Psychological Assessment Resources: (2012).

[19] Estes A Munson J Rogers SJ Greenson J Winter J Dawson G. Long-term outcomes of early intervention in 6-year-old children with autism Spectrum disorder. J Am Acad Child Adolesc Psychiatry. (2015) 54:580–7. doi: 10.1016/j.jaac.2015.04.005, PMID: 26088663 PMC4475272

[20] Iadarola S Levato L Harrison B Smith T Lecavalier L Johnson C. Teaching parents behavioral strategies for autism Spectrum disorder (ASD): effects on stress, strain, and competence. J Autism Dev Disord. (2018) 48:1031–40. doi: 10.1007/s10803-017-3339-2, PMID: 28988339

[21] National Institute for Health and Care Excellence. (2013). Autism Spectrum Disorder in Under 19s: Support and Management. Available at: https://www.nice.org.uk/guidance/cg170/chapter/Recommendations#interventions-for-behaviour-that-challenges 34283415

[22] Cravero C Guinchat V Claret-Tournier A Sahnoun C Bonniau B Bodeau N. Traitements médicamenteux reçus par les enfants, adolescents et jeunes adultes avec trouble du spectre autistique en France: Un état des lieux basé sur l’expérience parentale. Neuropsychiatr Enfance Adolesc. (2017) 65:33–41. doi: 10.1016/j.neurenf.2016.10.002

