# Supplementary material for: Clinical predictors of psychotropic medication prescription in children with ASD of the ELENA cohort
Source: Front Psychiatry. 2023 Jul 21;14:1153543. doi: 10.3389/fpsyt.2023.1153543 (PMC10400887; doi:10.3389/fpsyt.2023.1153543)
Supplement: Supplementary file 1 [file Table_1.docx]

**Supplementary file**

**Supp – Table 1**

Comparison of characteristics of the populations who responded to the question about taking psychotropic medication at V0 (YES versus NO) or did not respond (VM).

| **Taking medication** | | **n** | **Mean** | **SD** | **p-value** | **Post-hoc test^***^** |
| --- | --- | --- | --- | --- | --- | --- |
| Sex Male/ female | NO | 390/88 | - | - | 0.376* |  |
|  | YES | 144/33 | - | - |  |  |
|  | VM | 196/33 | - | - |  |  |
| Best QD V0 | NO | 420 | 73.64 | 27.54 | 0.053** | NO ≠ VM |
|  | YES | 162 | 71.51 | 30.43 |  |  |
|  | VM | 213 | 68.08 | 27.94 |  |  |
| Age V0 | NO | 478 | 5.77 | 3.23 | 0.002** | YES ≠ NO or VM |
|  | YES | 177 | 6.79 | 3.71 |  |  |
|  | VM | 229 | 5.59 | 3.13 |  |  |
| ADOS Comparison score  V0 | NO | 399 | 7.02 | 2.03 | 0.914** |  |
|  | YES | 154 | 6.96 | 1.84 |  |  |
|  | VM | 202 | 7.04 | 1.95 |  |  |
| VABS Communication V0 | NO | 464 | 71.60 | 14.94 | 0.0001** | NO ≠ YES or VM |
|  | YES | 171 | 67.82 | 16.28 |  |  |
|  | VM | 222 | 67.24 | 14.71 |  |  |
| VABS Socialisation V0 | NO | 464 | 71.26 | 10.64 | 0.0001** | NO ≠ YES or VM |
|  | YES | 171 | 66.16 | 10.66 |  |  |
|  | VM | 222 | 69.14 | 10.29 |  |  |
| VABS Daily living skills V0 | NO | 464 | 75.07 | 12.21 | 0.0001** | NO ≠ YES or VM |
|  | YES | 171 | 69.43 | 13.99 |  |  |
|  | VM | 223 | 72.22 | 13.07 |  |  |

* Chi-squared test (X^2^); ** Kruskall-Wallis test; ^***^: a Bonferroni correction was applied for 2-by-2 comparisons
